# Autophagy modulation attenuates sorafenib resistance in HCC induced in rats

**DOI:** 10.1038/s41419-024-06955-5

**Published:** 2024-08-16

**Authors:** Yomna Elleithi, Amal El-Gayar, Mohamed N. Amin

**Affiliations:** 1 Biochemistry Department, Faculty of Pharmacy, Mansoura University, Mansoura, 35516 Egypt; 2 Biochemistry Department, Faculty of Pharmacy, Mansoura National University, Gamasa, 7731168 Egypt

**Keywords:** Cancer microenvironment, Cancer therapeutic resistance

## Abstract

Hepatocellular carcinoma (HCC) has risen as the villain of cancer-related death globally, with a usual cruel forecasting. Sorafenib was officially approved by the FDA as first-line treatment for advanced HCC. Despite the brilliant promise revealed in research, actual clinical results are limited due to the widespread appearance of drug resistance. The tumor microenvironment (TME) has been correlated to pharmacological resistance, implying that existing cellular level strategies may be insufficient to improve therapy success. The role of autophagy in cancer is a two-edged sword. On one hand, autophagy permits malignant cells to overcome stress, such as hypoxic TME and therapy-induced starvation. Autophagy, on the other hand, plays an important role in damage suppression, which can reduce carcinogenesis. As a result, controlling autophagy is certainly a viable technique in cancer therapy. The goal of this study was to investigate at the impact of autophagy manipulation with sorafenib therapy by analyzing autophagy induction and inhibition to sorafenib monotherapy in rats with HCC. Western blot, ELISA, immunohistochemistry, flow cytometry, and quantitative-PCR were used to investigate autophagy, apoptosis, and the cell cycle. Routine biochemical and pathological testing was performed. Ultracellular features and autophagic entities were observed using a transmission electron microscope (TEM). Both regimens demonstrated significant reductions in chemotherapeutic resistance and hepatoprotective effects. According to the findings, both autophagic inhibitors and inducers are attractive candidates for combating sorafenib-induced resistance in HCC.

## Introduction

**Fig. 1:**
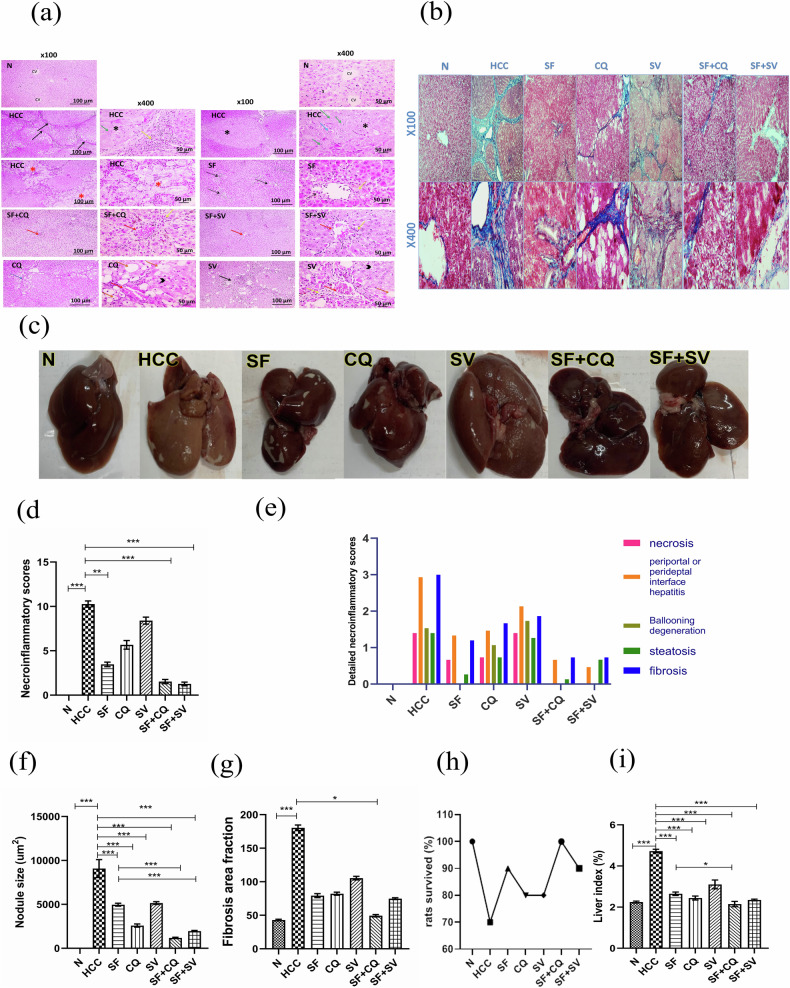
Histopathology, fibrosis and survival rates. **a** Representative microscopic images of H&E stained hepatic sections from control (N), hepatocellular carcinoma (HCC), and HCC treated with sorafenib (SF), sorafenib + simvastatin (SF + SV), sorafenib + hydroxychloroquine (SF + CQ), hydroxychloroquine (CQ), or simvastatin (SV) groups; central vein (CV), sinusoids (s), necroinflammatory zones (black arrows), leukocytes and hemosiderin laden macrophages (yellow arrow), well‐differentiated HCC (*), enlarged rounded vesicular nuclei (green arrow), cytoplasmic eosinophilic inclusions (blue arrow), cholangiocarcinoma (*), necrotic cells (white arrows), congested blood vessels (red arrows), steatosis (arrowheads), ballooning degeneration (orange arrows), centrilobular areas (dashed blue arrows), fibrosis around central veins (dashed black arrows), low magnification X: 100 bar and high magnification X: 400 bar. **b** Representative microscopic images of Masson Trichome stained liver sections; upper panel X 100 and lower panel X 400. **c** Liver gross examination; **d** Histopathological necroinflammatory scores; **e** Detailed histopathological necroinflammatory scores; **f** Mean diameter of tumor nodules; **g** Mean fibrosis area fraction using masson trichome staining; **h** Percentage survival of rats; **i** Liver index at the end of the study in different groups; *p < 0.05; **p < 0.01; ***p < 0.001, using one-way analysis of variance (ANOVA) followed by Tukey’s post-hoc test (**f**–**i**), and Kruskal–Wallis test followed by Dunn’s post-hoc test (**d**).

Hepatocellular carcinoma (HCC) is a leading cause of cancer-related death worldwide. Hepatic cancer is sixth in terms of incidence and second in terms mortality among men worldwide. The World Health Organization forecasts that more than one million individuals would die from liver cancer by 2030, based on annual figures [1]. Reasonable advances have been developed for prevention, surveillance, early detection, diagnosis, and treatment [2]. Treatment medications for advanced HCC were not avilable until 2006. Chemotherapeutic agents (cisplatin, doxorubicin, epirubicin, etc.), immunotherapy (interferon), hormonal drugs, and many more medications provided unsatisfactory or negative outcomes [3].

Animal models are important tools in cancer research. A variety of animal models have been established to better understand the pathophysiology of HCC and the impact of prospective therapy. Several morphological, histogenic, and biochemical aspects of human HCC are shared by TAA induced hepato-carcinogenesis in animals, which begins with an irreversible alteration of DNA structure, leading to HCC subsequent to liver cirrhosis, which resembles the human HCC [4, 5].

Sorafenib (Nexavar®) is the singular systemic agent used in advanced HCC that is approved by the Food and Drug Administration. Based on the results of Phase III trials of Sorafenib Asia-Pacific and pivotal Sorafenib Hepatocellular carcinoma Assessment Randomized Protocol trials in advanced HCC patients with Child–Pugh class A, it was approved in the European Union and the USA in 2007 [6, 7]. Sorafenib is a multikinase inhibitor which is administered orally [8–10]. Sorafenib decreased the proliferation of HCC cells, tumor growth and angiogenesis, in addition to inducing their apoptosis, in in vitro and animal models of HCC [11].

Despite these impressive results, sorafenib conferred only limited benefits to patients with advanced HCC and failed to completely cure them. It was beneficial to barely about 30% of the patients, and the presence of primary and acquired resistance to sorafenib was detected in HCC cells [6, 7]. A considerable number of patients develop primary resistance—a result of HCC genetic heterogeneity—but in the majority of the cases, the resistance is acquired [12]. This is seen as a compensatory consequence of continuous drug exposure. Numerous mechanisms are involved in reducing responsiveness to treatment with sorafenib. These mechanisms include phosphoinositide 3-kinase (PI3K)/protein kinase B (Akt) and Janus kinase-signal transducer and activator of transcription (JAK-STAT) pathways, deactivation of pro-apoptotic signals, cancer stem cells, epithelial–mesenchymal transition and hypoxia-inducible response [13, 14].

Autophagy (meaning “self-eating”) is the process responsible for bulk degradation of long-lived cytoplasmic proteins and organelles. Macro-autophagy is characterized by the capturing and sequestration of the target cargo far from the lysosome, unlike micro-autophagy and chaperone-mediated autophagy. Macro-autophagy involves the de novo synthesis of double-membraned autophagic vesicles, called autophagosomes, which capture cargo and then translocate it to the lysosome [15]. Autophagy is basically a cytoprotective mechanism; but autophagic dysfunction is correlated with a number of human pathologies, such as lung, heart, and liver disease, aging, neurodegeneration, myopathies and diabetes [16]. Recently, it has been reported that autophagy defects are linked to tumorigenesis. It has also been shown that autophagy is involved in both the promotion and suppression of tumorigenesis [17].

The objective of our study was to investigate the effect of coupling sorafenib with autophagy modulators on the treatment efficiency of HCC. We evaluated the efficacy of a treatment strategy involving either sorafenib only (SF), sorafenib in combination with simvastatin, an autophagy inducer, (SF + SV) or sorafenib in combination with hydroxychloroquine, an autophagy inhibitor, (SF + CQ), in hepatocellular carcinoma rat model that resembles human HCC. To accelerate the transition into clinical application, the compounds we used were drugs already in use for other indications in humans for years and have their safety profile proven.

## Results

### Sorafenib combination with autophagic modulators improved hepatic function and suppressed HCC progression more effectively

#### H&E histopathological examination, nodule size and necroinflammatory scoring

Microscopic images of H&E-stained hepatic sections from the N group showed normal arrangement of hepatic cords around the central vein with normal portal areas and sinusoids (Fig. 1a). H&E-stained hepatic sections from the HCC group showed loss of normal hepatic architecture due to arrangement of hepatocytes into solid nodules and the central vein surrounded by thick necroinflammatory zones containing fibrous tissue infiltrated with leukocytes and hemosiderin-laden macrophages. Some nodules developed well‐differentiated HCC. Cells of HCC are polygonal with distinct cell membranes, an eosinophilic granular cytoplasm, enlarged rounded vesicular nuclei with coarse chromatin, thickened nuclear membrane and prominent nucleoli without mitotic figures and cytoplasmic eosinophilic inclusions. Cholangiocarcinoma appears in some sections (Fig. 1a).

H&E-stained liver sections from the SV, CQ, and SF groups exhibited partial to marked improvements in hepatic architecture. The SF group showed perivascular mild fibrosis around central veins with the presence of a few necrotic hepatocytes and leukocytes (Fig. 1a). In addition, the CQ group showed decreased degrees of inflammation, necrosis and congestion in centrilobular areas with the presence of micro-vesicular steatosis and ballooning degeneration in hepatocytes around centrilobular areas (Fig. 1a).

Moreover, the SV group showed centrilobular thick necroinflammatory areas, containing necrotic cells, leukocytes, hemosiderin-laden macrophages and congested blood vessels, and surrounded by multifocal micro-or macro-vesicular steatosis to ballooning degeneration (Fig. 1a). On the other hand, hepatic sections from treated group SF + SV and SF + CQ showed marked improvement in hepatic architecture with mildly congested blood vessels and very few leukocytic cells infiltrating (Fig. 1a).

The combination treatment SF + CQ and SF + SV groups significantly reduced the necroinflammatory score by 6–8 folds compared to that of the HCC group (Fig. 1d, e). Also, the necroinflammatory score in these groups was non-significantly lower than that of the SF group alone. Consistently, the mean tumor nodule size was significantly lower in SF + CQ and SF + SV groups compared to that of HCC or SF groups (Fig. 1f).

#### Masson’s trichrome staining and fibrosis area fraction

Normal control group (N) showed the normal minimal distribution of the bluish stained collagen fibers around the central veins. HCC group appeared with widespread extension of the bluish stained collagen fibers from the central vein vicinity to invade in between the hepatocytes (Fig. 1b). SF group showed apparently less distribution of collagen bundles than the HCC group. CQ and SV groups also had less prominent collagen than the HCC group. SF + CQ and SF + SV groups exhibited the least collagen content. The fibrotic area fraction was significantly lower in SF + CQ group compared with HCC group (Fig. 1g).

#### Liver gross examination, Liver index and Overall survival

Livers isolated from HCC group showed a slightly rough nodular surface with noticeable faint discoloration compared to reddish and smooth surfaces of (N) livers. On the other hand, all the treatment groups showed a decrease in the nodular surface and darker colors specially in SF + CQ group, rendering them smoother and reddish when compared to HCC group (Fig. 1c). likewise, the liver index was significantly lower in the SF + CQ group compared with either SF group (*p* < 0.05) or HCC group (*p* < 0.001) (Fig. 1i). Also, the overall survival over the course of 3-weeks treatment was the highest in SF + CQ (100%), similar to N group, (Fig. 1h).

#### TEM histopathological micrographs

**Fig. 2:**
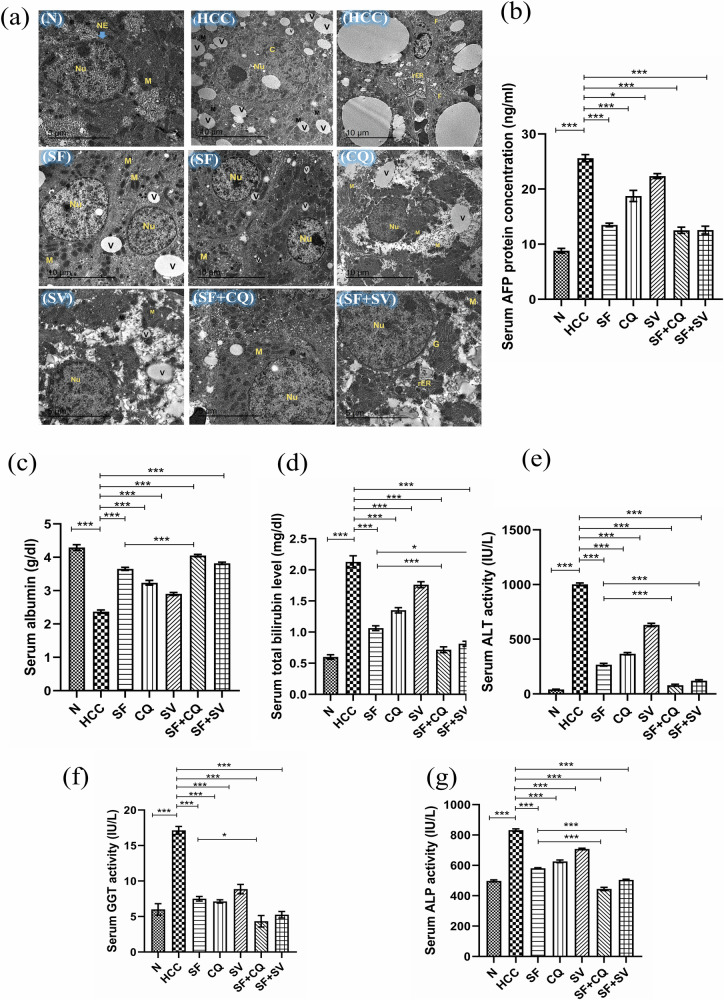
TEM histopathology, AFP & liver function tests. **a** Transmission electron microscope (TEM) images of ultracellular structures in control (N), hepatocellular carcinoma (HCC), and HCC treated with sorafenib (SF), sorafenib + simvastatin (SF + SV), sorafenib + hydroxychloroquine (SF + CQ), hydroxychloroquine (CQ), or simvastatin (SV) groups; nuclear envelope (NE), nucleus (Nu), and mitochondria (M), vacuoles (V), rough endoplasmic reticulum (rER), infiltrated fat droplets (F). **b**–**d** Serum protein levels of **b** alpha-fetoprotein (AFP); **c** albumin; and **d** total bilirubin at the end of the study in different groups. **e**–**g** Serum activity of (**e**) alanine aminotransferase (ALT); **f** gamma glutamyl transferase (GGT); and **g** alkaline phosphatase (ALP) at the end of the study in different groups; *p < 0.05; **p < 0.01; ***p < 0.001, using one-way analysis of variance (ANOVA) followed by Tukey’s post-hoc test.

HCC group hepatic cells showed disintegrated cellular content, abnormal mega-mitochondria, pyknotic nucleus, with abnormal chromatin distribution, very high content of vacuoles, breakdown of rough endoplasmic reticulum and infiltrated fat droplets, (Fig. 2a). In addition, SF group hepatic cells showed more integrated cellular content, nuclear, and mitochondrial structures and lower content of vacuoles in comparison to HCC group, (Fig. 2a).

CQ and SV groups showed progressing degeneration of hepatic cells and less integrated organelles or abnormal mitochondria in addition to numerous vacuoles, (Fig. 2a).

TEM images showed ameliorative effects of the combination treatment in SF + CQ and SF + SV groups when compared to HCC or SF groups. SF + CQ and SF + SV groups showed ameliorative architecture of hepatic cells and more integrated organelles indicating regeneration, (Fig. 2a).

#### Serum AFP and routine hepatic function tests

Serum AFP level was lowered significantly (*P* < 0.001) by either SF monotherapy or SF combinations compared to HCC group, but there was no statistical significance between SF and SF + CQ & SF + SV, (Fig. 2b). Additionally, the synthetic function of liver evidenced by albumin serum concentration was less affected in case of SF + CQ compared to SF group (Fig. 2c). Similarly, the detoxification function evidenced by serum bilirubin was significantly less impaired compared to SF group, indicating more successful treatment of hepatobiliary disease (Fig. 2d). Serum ALT, GGT, and ALP activities were significantly lower in SF + CQ compared to SF group. consistently, Serum ALT and ALP activities were significantly lower in SF + SV compared to SF group (Fig. 2e–g).

### Success of autophagic modulation by hydroxychloroquine and simvastatin

#### Western blotting of LC3 and its relative gene expression using qPCR

**Fig. 3:**
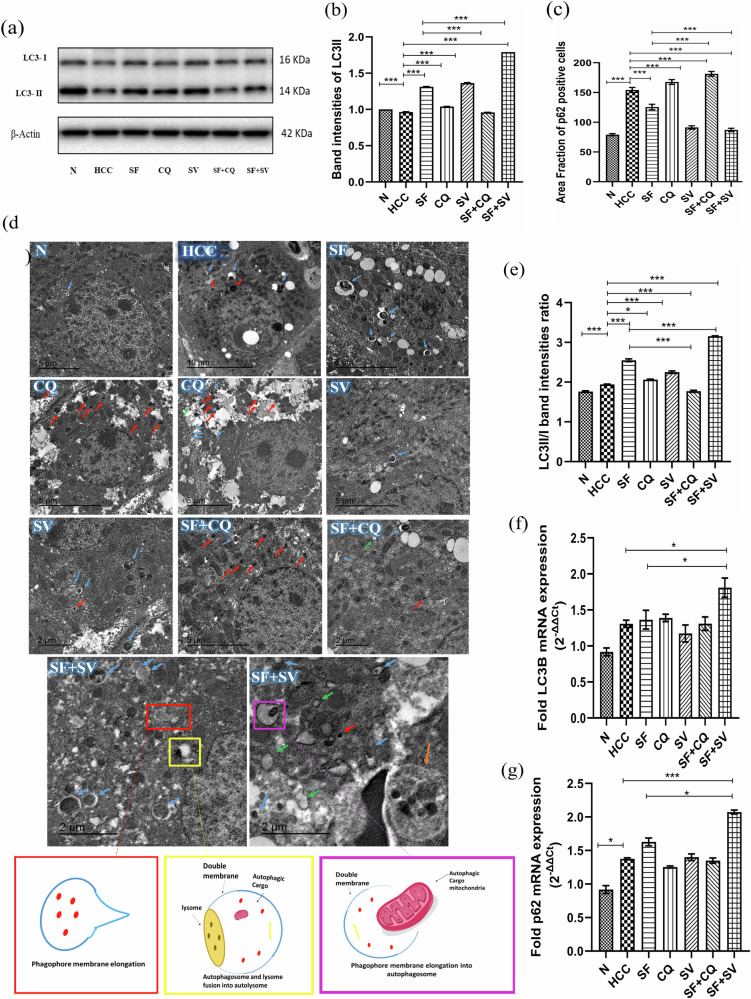
Autophagy assessment. **a** Western blotting analysis of LC3II/I in liver protein lysate at the end of the study in control (N), hepatocellular carcinoma (HCC), and HCC treated with sorafenib (SF), sorafenib + simvastatin (SF + SV), sorafenib + hydroxychloroquine (SF + CQ), hydroxychloroquine (CQ), or simvastatin (SV) groups. **b** Western blotting band intensities of LC3II normalized to β-actin; **c** Mean area fraction of p62 positive cells in liver sections; **d** Transmission electron microscope (TEM) images of autophagy related cellular structures; lysosomes (red arrows), autophagosomes (green arrows), autolysomes (blue arrows), giant autolysosome (orange arrow); **e** Ratio of LC3II/LC3I western blot band intensities; Relative mRNA expression (2^-ΔΔct^) of (**f**) LC3B and (**g**) p62 normalized to GAPDH at the end of the study in different groups; *p < 0.05; **p < 0.01; ***p < 0.001, using one-way analysis of variance (ANOVA) followed by Tukey’s post-hoc test.

In LC3 blots, the upper band is LC3-I (visible molecular weight: 16 kDa), whereas the bottom band is LC3-II (visible molecular weight is 14 kDa), (Fig. 3a).

The blot showed rise in LC3II/I band intensity in the HCC group above that of N group (*p* < 0.001), (Fig. 3a, b) manifesting induction of autophagy in HCC group. Similarly, LC3-II was elevated expectedly in either SF, SV or CQ when compared to HCC (*p* < 0.001).

The induction of autophagy by simvastatin drug was indicated by the high LC3II and LC3II/I band intensities in both of SV and SF + SV groups when compared with the HCC group (*p* < 0.001) and the SF group (*p* < 0.001). Additionally, SF + SV had significant increase in LC3-II when compared to SF (*p* < 0.001). The exception was SF + CQ, which showed a relative weak LC3II band than what was anticipated, (Fig. 3a, b).

We then validated the protein expression with gene expression via mRNA quantification of LC3B, (Fig. 3f).

#### Immunohistochemistry of p62 and its relative gene expression using qPCR

There was no significant variation in p62 gene expression between most of the study groups (Fig. 3g); indicating that the variation in p62 levels in most groups relies mainly on the rate of autophagic degradation. The exceptions were a significant increase in p62 mRNA expression in HCC compared to N (*p* < 0.05), and in SF + SV group compared to SF group (*p* < 0.05). During excessive autophagy, the transcriptional activation of lysosomal and autophagic genes should be adequate to compensate for the depletion of the corresponding proteins [18], as clearly seen in SF + SV group.

The p62 protein expression confirmed success of autophagy inhibition and induction (Fig. 3c).

**Fig. 4:**
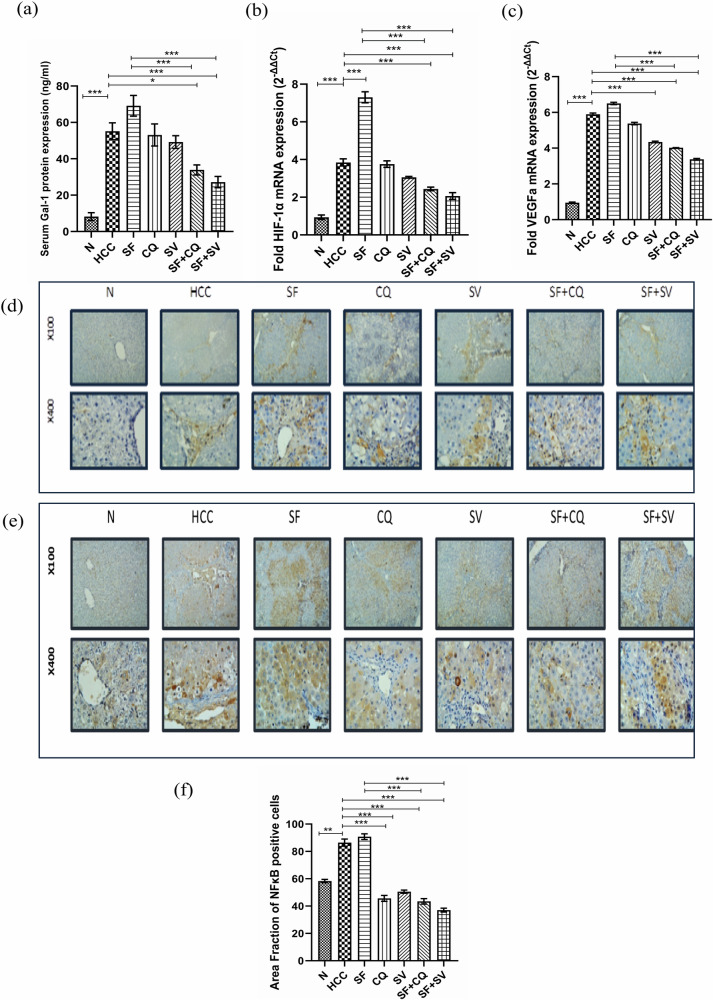
Sorafenib resistance assessment. **a** Mean serum protein levels of Galectin -1 (Gal-1) at the end of the study in control (N), hepatocellular carcinoma (HCC), and HCC treated with sorafenib (SF), sorafenib + simvastatin (SF + SV), sorafenib + hydroxychloroquine (SF + CQ), hydroxychloroquine (CQ), or simvastatin (SV) groups. Relative mRNA expression (2^−ΔΔct^) of (**b**) hypoxia inducible factor-1α (HIF-1α) and (**c**) vascular endothelial growth factor-a (VEGFa) normalized to GAPDH at the end of the study in different groups. Representative microscopic images of liver sections stained against (**d**) nuclear factor kappa-B (NF-κB), and (**e**) p62 protein (brown color) at the end of the study in different groups counterstained with hematoxylin; upper panel: x100 bar; lower panel: x400 bar. **f** Mean area fraction of NF-κB positive cells in liver sections at the end of the study in different groups; *p < 0.05; **p < 0.01; ***p < 0.001, using one-way analysis of variance (ANOVA) followed by Tukey’s post-hoc test.

A significant increase in p62 protein in HCC group compared to N group (*p* < 0.001) was observed (Fig. 4e). Then, the significant increase of p62 mRNA in HCC group compared to N group (*p* < 0.05) confirmed the high level of autophagic activation.

The autophagic induction in SF was manifested in the form of a decrease in p62 protein expression (*p* < 0.001) with no significant change in p62 mRNA level, when compared with the results for HCC. Together with a significant increase in LC3II and LC3II/I ratio (*p* < 0.0001), this data confirmed that the decrease in p62 protein was due to autophagic degradation of p62 and not due to transcriptional changes.

The protein expression of p62 showed elevated levels in SF + CQ when compared to SF (*p* < 0.001) (Fig. 4e), with no significant change in mRNA levels (Fig. 3g), confirming autophagy inhibition by hydroxychloroquine.

The expression of p62 protein came in accordance with LC3-II/LC3-I (Fig. 3e) emphasizing the success of autophagic modulation in SF + CQ & SF + SV when compared to SF, (*p* < 0.001) in both cases.

#### TEM micrographs of ultra-thin liver sections of autophagy related cellular structures

Photos from TEM showed very few autophagic bodies in N group (Fig. 3d), and notably higher presence of lysosomes and autolysosomes (AL) in HCC group. AL were significantly more present in liver sections from SF group. On the other hand, photos from CQ group were characterized by heavy accumulation of lysosomes. SV group showed presence of AL in a manner parallel to that of SF. Combinations groups showed accumulation of lysosomes with the presence of some AL in case of SF + CQ, and accumulation of AL and autophagosomes in case of SF + SV.

Notably, the high induction of autophagy in case of SF + SV allowed the visualization of various stages of autophagy as phagophore elongation, autophagosome formation and autophagosome lysosomal fusion into AL, illustrated and simplified below the TEM photos from SF + SV. A giant AL was also visualized in photos from the same group.

### Autophagic modulators combination with Sorafenib sensitized HCC to sorafenib

#### Autophagy modulation decreased sorafenib resistance as indicated upon measuring Serum Galectin-1 level by ELISA

We found a significant increase in Galectin-1 (Gal-1) serum levels in HCC group than N group (*p* < 0.001), and a slight non-significant increase with SF when compared to HCC. But in the combination therapy, SF + CQ and SF + SV Gal-1 was decreased when compared to HCC, (*p* < 0.05) and (*p* < 0.001), respectively. Interestingly, a significantly lower level of Gal-1 was seen in SF + CQ & SF + SV in comparison to SF (*p* < 0.001) (Fig. 4a).

#### Relative gene expression of HIF-1α and VEGFa by qPCR

To further elucidate transcriptional changes associated with sorafenib treatment, we measured the mRNA relative expression of Hypoxia Inducible factor-1 (HIF-1α), and found Significant increase in HIF-1α in HCC group to about 4-fold that of N group (*p* < 0.001), and further increased significantly in SF group (*p* < 0.001) indicating the occurrence of sorafenib resistance. The occurrence of resistance was also confirmed by results of VEGFa gene expression which was significantly lowered by SF + CQ & SF + SV too, as in case of HIF-1α, in contrast to SF, illustrated in (Fig. 4b, c).

#### Immunohistochemistry of nuclear factor kabba B (NF-κB)

Sorafenib resistance was detected as well by the high protein expression of NF-κB in Sorafenib monotherapy. Nevertheless, NF-κB decreased in combinations with either simvastatin or hydroxychloroquine (Fig. 4d, f).

### Apoptosis induction and cell cycle arrest

#### Hydroxychloroquine and simvastatin synergically increased caspase-3 immunohistochemistry and its apoptotic index with sorafenib

**Fig. 5:**
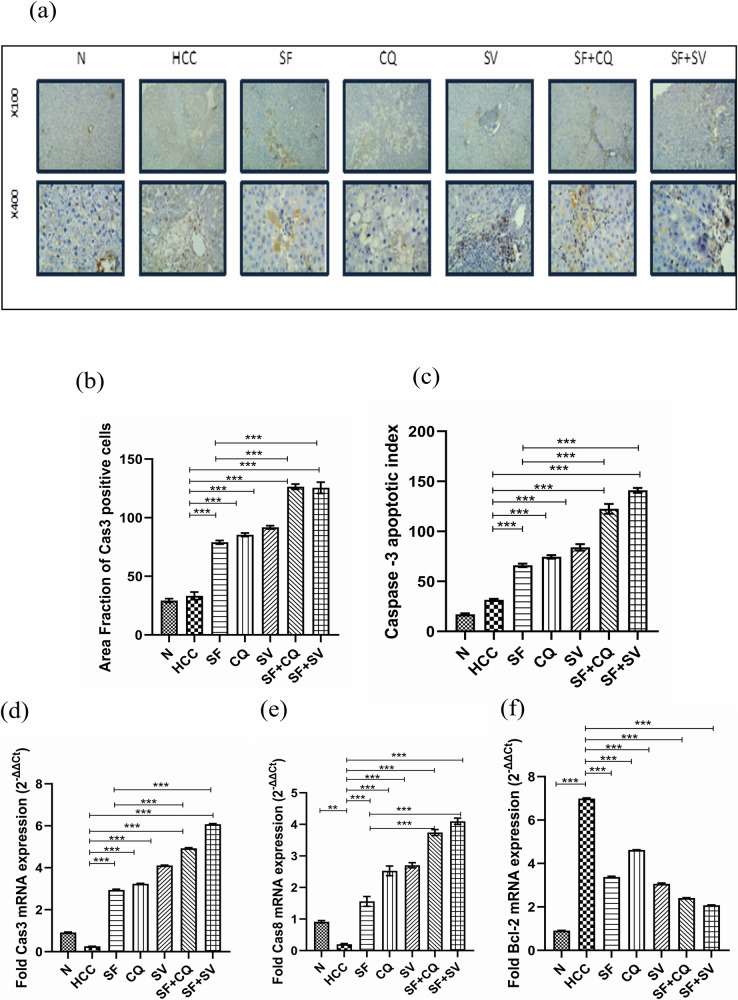
Apoptosis induction. **a** Representative microscopic images of liver sections stained against active caspase-3, counterstained with hematoxylin at the end of the study in control (N), hepatocellular carcinoma (HCC), and HCC treated with sorafenib (SF), sorafenib + simvastatin (SF + SV), sorafenib + hydroxychloroquine (SF + CQ), hydroxychloroquine (CQ), or simvastatin (SV) groups; upper panel: x100 bar; lower panel: x400 bar. **b** Mean area fraction of caspase-3 (cas-3) positive cells in liver sections; **c** caspase-3 apoptotic index at the end of the study in different groups. **d**–**f** Relative mRNA expression (2^−ΔΔct^) of (**d**) cas-3; (**e**) caspase-8 (cas-8); (**f**) Bcl_2_ normalized to GAPDH at the end of the study in different groups; *p < 0.05; **p < 0.01; ***p < 0.001, using one-way analysis of variance (ANOVA) followed by Tukey’s post-hoc test.

Caspase-3 (Cas-3) protein levels in liver were analyzed to determine the apoptosis activation. As shown in (Fig. 5a–c), autophagic modulators in combination with sorafenib elevated active cas-3 protein expression, and hence, had high apoptotic index. We further confirmed these results by qPCR and cell cycle analysis.

#### Hydroxychloroquine and simvastatin synergically increased apoptosis on the transcriptional level

We found that HCC on the gene level was downregulating the expression of caspases and upregulating the Bcl2 (B-cell lymphoma 2). While these levels were reversed on the combination groups (Fig. 5d–f).

#### Hydroxychloroquine and simvastatin synergically increased extrinsic pathway of apoptosis via upregulation of the cellular death receptors

**Fig. 6:**
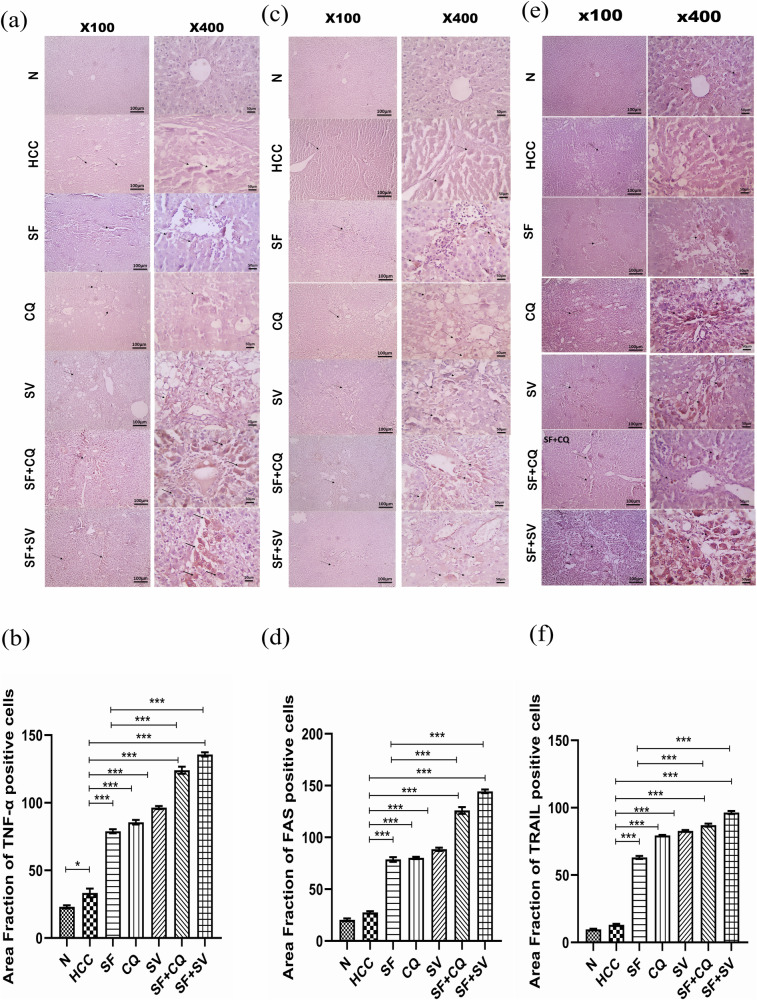
Immunostaining of death receptors. **a** Representative microscopic images of liver sections stained against Tumour Necrosis Factor alpha (TNF-α), counterstained with hematoxylin at the end of the study in control (N), hepatocellular carcinoma (HCC), and HCC treated with sorafenib (SF), sorafenib + simvastatin (SF + SV), sorafenib + hydroxychloroquine (SF + CQ), hydroxychloroquine (CQ), or simvastatin (SV) groups; left panel: x100 bar 100 µm; right panel: x400 bar 50 µm. **b** Mean area fraction of TNF-α positive cells in liver sections; **c** Representative microscopic images of liver sections stained against FAS, counterstained with hematoxylin at the end of the study; left panel: x100 bar 100 µm; right panel: x400 bar 50 µm. **d** Mean area fraction of FAS positive cells in liver sections. **e** Representative microscopic images of liver sections stained against TNF-Related Apoptosis-Inducing Ligand (TRAIL), counterstained with hematoxylin at the end of the study in control (N), hepatocellular carcinoma (HCC), and HCC treated with sorafenib (SF), sorafenib + simvastatin (SF + SV), sorafenib + hydroxychloroquine (SF + CQ), hydroxychloroquine (CQ), or simvastatin (SV) groups; left panel: x100 bar 100 µm; right panel: x400 bar 50 µm. **f** Mean area fraction of TRAIL positive cells in liver sections; *p < 0.05; **p < 0.01; ***p < 0.001. using one-way analysis of variance (ANOVA) followed by Tukey’s post-hoc test.

Microscopic pictures of the immunostained hepatic sections against Tumor Necrosis Factor-alpha (TNFα) from control group (N) showed negatively stained hepatocytes. Few positively stained hepatocytes are seen in hepatic sections from HCC group (Fig. 6a). The higher staining was statistically significant (*p* < 0.05), (Fig. 6b).

But we noticed an increased number of positively stained hepatocytes in SF group and few ones in CQ group (Fig. 6a). There was also much higher number of positively stained hepatocytes are seen in hepatic sections from SV group (thin arrows). Highly significant increase of numbers of positively stained hepatocytes was seen in hepatic sections from of treated group SF + CQ and SF + SV (*p* < 0.001) when compared to the untreated HCC group (Fig. 6a, b). The same was seen in microscopic pictures of the immunostained hepatic sections against apoptosis antigen 1, FAS receptors (also known as CD95/APO1). The extrinsic apoptotic death receptors were significantly higher in SF + CQ & SF + SV groups when compared to SF group (*p* < 0.001) (Fig. 6d), as indicated by the thin arrows in microscopic pictures (Fig. 6c).

When it comes to TNF-Related Apoptosis-Inducing Ligand (TRAIL) immunostaining of liver sections similarly showed significant superiority of autophagic modulators combinations over sorafenib alone (Fig. 6e). A statistically significant elevation in positively stained hepatocytes was seen in hepatic sections from SF + CQ and SF + SV groups (*p* < 0.001) when compared to the untreated HCC group and SF group (Fig. 6e, f)

#### Sorafenib arrested cell cycle at G0/1 in its monotherapy and in G2/M when combined with Hydroxychloroquine and simvastatin

**Fig. 7:**
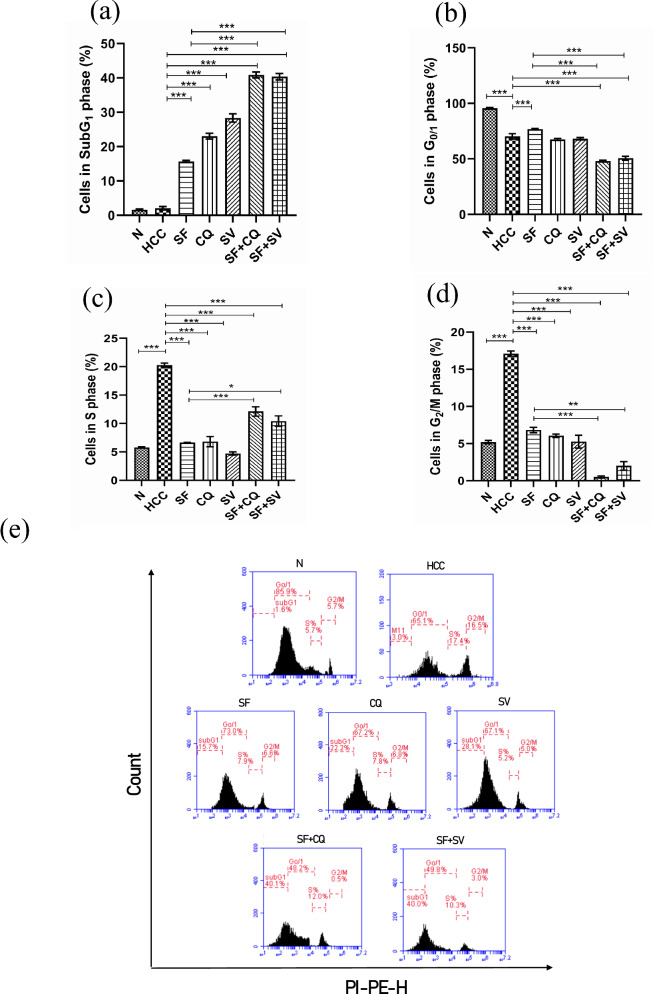
Cell cycle analysis. Statistical representation of cell cycle analysis results for **a** subG_1_ phase; **b** G_0_/G_1_ phase; **c** S phase; and **d** G2/M phase at the end of the study in control (N), hepatocellular carcinoma (HCC), and HCC treated with sorafenib (SF), sorafenib + simvastatin (SF + SV), sorafenib + hydroxychloroquine (SF + CQ), hydroxychloroquine (CQ), or simvastatin (SV) groups. **e** Representative figure from cell cycle analysis charts at the end of the study in different groups; *p < 0.05; **p < 0.01; ***p < 0.001, using one-way analysis of variance (ANOVA) followed by Tukey’s post-hoc test.

Cell cycle analysis results (Fig. 7), showed significant elevation in necrosis or apoptosis appearing as SubG1 phase in SF, CQ & SV (*p* < 0.001), when compared to HCC (Table 1).

**Table 1:** Cell cycle analysis results (mean % of cells ± S.E.M).

	*N*	HCC	SF	CQ	SV	SF + CQ	SF + SV
SubG1	1.65 ± 0.21	2.05 ± 0.49	15.71 ± 0.27	23.08 ± 0.83	28.35 ± 1.20	40.88 ± 0.85	40.38 ± 0.95
G0/1	95.63 ± 0.69	70.30 ± 0.35	76.85 ± 0.45	67.55 ± 0.92	68.20 ± 1.05	48 ± 0.82	50.65 ± 1.66
S	5.78 ± 0.11	20.28 ± 0.37	6.62 ± 0.05	6.80 ± 0.90	4.73 ± 0.28	12.13 ± 0.83	10.43 ± 0.91
G2M	5.20 ± 0.23	17.10 ± 0.36	6.82 ± 0.30	6.05 ± 0.23	5.25 ± 0.85	0.50 ± 0.15	2 ± 0.58

Percentage of cells of Normal control (N), hepatocellular carcinoma (HCC), and HCC treated with sorafenib (SF), sorafenib + simvastatin (SF + SV), sorafenib + hydroxychloroquine (SF + CQ), hydroxychloroquine (CQ), or simvastatin (SV) groups in each phase.

Interestingly, that increase was higher in SF + CQ & SF + SV than SF of sorafenib (*p* < 0.001), (Fig. 7a).

We also noticed that SF caused G0/1 cell cycle, (Fig. 7b). On the contrary, the combination regimens, SF + CQ & SF + SV, both caused cell cycle arrest at G2/M phase, marked by decrease of the cells at the active mitosis stage (M phase) and their stop at the DNA synthesis phase (S phase) with inability to complete the division for replication, (Fig. 7c, d). Representative figures from each study group results are shown on (Fig. 7e).

## Discussion

Sorafenib is a promising therapeutic drug for HCC, however optimal results have not been achieved, yet. The development of acquired resistance after treatment has also piqued the interest of researchers [9, 14].

Autophagy is one of the pathways that is both activated by sorafenib and implicated in its acquired resistance [19]. Different autophagy responses in different HCC models showed various sensitivities to sorafenib [20]. Several studies, covered in the review by Sun et al. [19], have showed a synergistic antitumor effect arising from the modulation of autophagy during sorafenib treatment, with autophagic inhibitors, such as chloroquine/hydroxychloroquine [21, 22] as well as direct autophagy inducers such as metformin [23]. In addition, excess stimulation of autophagy may also result in cell death rather than survival [24]. Hence, the result of autophagy in tumor cells might be cell mortalitiy or survival [25]. Essentially, restoration of physiological level of autophagy might be one of the most important strategies for avoiding cellular resistance to the antineoplastic drug [26, 27].

The autophagic alterations and success of autophagy modulation were assessed by using protein and gene levels of p62 and LC3, collectively with TEM visualization of lysosomes, autophagosome and autolysosomes. The expression of p62 protein came in accordance with LC3-II/LC3-I in addition to autophagic bodies and lysosomes visualization in ultrathin sections of liver under TEM. These sets of data emphasized the success of autophagic modulation in SF + CQ & SF + SV when compared to SF.

Oxygen deprivation is frequent in solid tumors, including HCC, and promotes VEGF synthesis and angiogenesis via HIF-1α stimulation [28]. Hence, sorafenib decreases angiogenesis via the inhibition of the HIF-1α/VEGF signaling [29]. The inhibition of angiogenesis leads to hypoxia and the survival of the aggressive resistant cells. Consequently, hypoxia has been linked to acquired sorafenib resistance. This scenario reduces the efficacy of sorafenib [30].

HIF-1α is a key regulator of VEGF expression. Under hypoxic conditions, accumulating HIF-1α increases VEGF [31]. Several studies have showed a compensatory response to sorafenib treatment by an increase in HIF-1α after an initial lowering [32, 33].

Liang et al. found that regular administration of sorafenib in HCC mice models increased HIF-1α and NF-κB transcriptional activity and protein expression. Accordingly, this might induce sorafenib resistance as a cytoprotective adaptive response. They also noted that HCC tissues from sorafenib-resistant patients exhibited higher intra-tumoral hypoxia and HIF-1α expression than sorafenib-sensitive or untreated HCC tissues [30].

Furthermore, NF-κB may stimulate HIF-1α and HIF-1α can control NF-κB. The occurrence of hypoxia and inflammation is a hallmark of cancer. The induction of NF-κB is a common characteristic of cancer cell responses to chemotherapy. Increased NF-κB activity, for example, has been linked to a poor response to neoadjuvant chemotherapy and radiation in patients with esophageal cancer [34]. Therefore, NF-κB may be a crucial indicator of drug resistance [35].

Several investigations have revealed that tumor cells subjected to hypoxia express greater amounts of Gal-1 [36]. Gal-1 can trigger epithelial–mesenchymal transition: a critical phase in the development of cancer in human cells, and a major pathway of sorafenib resistance [37], Multiple studies have reported the involvement of Gal-1 in metastatic potential, and the impact of Gal-1 knockdown on treatment sensitivity in HCC [38, 39], as well as other forms of cancer [40–42]. Sorafenib-resistant HCC models show increased Gal-1 levels, which may enhance cancer spread and tumor invasion. These data validated utilizing Gal-1 as a new HCC predictive and prognostic biomarker for sorafenib resistance [33].

To sum up all of these data, and to summarize the outstanding work done in the field of finding a proper prognostic tool for sorafenib resistance, the levels of HIF-1α, VEGFa, NF-κB and Gal-1 can define the cellular resistance state of cells to sorafenib treatment.

The results of our investigation on acquired sorafenib resistance showed these four biomarkers, increasing in the sorafenib monotherapy group. Nevertheless, there was a significant decrease in these biomarkers in the SF + SV and SF + CQ groups compared to HCC. The decrease was more prominent than that observed in SF group compared to HCC group. Such results imply that modulating autophagy prevented the development of resistance.

Apoptosis is strongly linked with cell cycle progress. This relationship plays an important function in neoplasia. Furthermore, carcinogenesis is linked to cell cycle regulation [43]. For more than three decades, the establishment of anti-tumor medicines and regimens that induce apoptosis has been a mainstay and aim of clinical oncology.

We used qPCR for analyzing combination therapy impact on Caspase-3 and Caspase-8. On top of that, the transcriptional level of anti-apoptotic Bcl2 was measured to examine intrinsic apoptotic pathway. On the other hand, extrinsic pathway of apoptosis was assessed by immunostaining of some death receptors such as TNF-α, TRAIL and CD95 (FAS).

upon contrasting the untreated HCC group, all treatment groups demonstrated a significant rise in early and late apoptotic signs. These findings are consistent with previous research that believed sorafenib [7, 44–46], simvastatin [47–50] and hydroxychloroquine [51–53] to induce apoptosis in cancer cells. Reasonably, the combination therapies showed a marked much higher elevation in apoptotic signs than that achieved by sorafenib alone (*p* < 0.0001) indicating its efficient anti-cancer effect.

We did not find a significant difference between the two sorafenib combinations in resistance biomarkers quantified (HIF-1α, VEGFa, NF-κB and Gal-1). But it was worth the mention that caspase-3 mRNA showed significant increase in the favor of SF + SV group, implicating slight superiority of simvastatin in inducing apoptosis compared to hydroxychloroquine.

Our study demonstrated that autophagy modulators induced G2/M cell cycle arrest and apoptosis leading to decreased sorafenib-resistance. Treatment with either SF, CQ, or SV increased accumulation of cells in G0/G1, which means that more cells have achieved a resting state (quiescence). Also, the combination therapies caused the cells to accumulate in S phase and they were unable to enter the mitotic stage.

Regarding hepatic hemostasis, the histopathological score of liver sections obtained from the combination treatments of sorafenib was way more improved than that of sorafenib alone, as well as the biochemical results of liver function tests and fibrosis staining.

There was a slight superiority of the hydroxychloroquine combination to the simvastatin one in the context of hepatic hemostasis, and this might be explained by the better hepatoprotective signs in CQ group compared to SV group. Such results are consistent with the previous studies that elucidated enhancement of liver indices in case of chloroquine treatment in TAA induced HCC rat models [54], and hydroxychloroquine in diabetic rats [55], liver steatosis [56] and in drug induced hepatotoxicity [57]. However, the improvement was not statistically significant compared to that of simvastatin combination except for alkaline phosphatase (*P* < 0.05).

It is worth mentioning that hydroxychloroquine showed beneficial effects on cancer regression in clinical studies of HCC patients (NCT03344172, NCT00969306, NCT01006369 & NCT01273805) [58]. Also, it showed beneficial effect in clinical trials of HCC patients-treated with sorafenib (NCT03037437). Those effects may not necessarily be connected with a blockade in autophagy [59, 60].

The immunostaining of apoptotic cell receptors and caspas-3 apoptotic index in addition to gene expression of Bcl-2 showed a remarkable privilege possessed by SF + SV group over SF + CQ group in terms of apoptotic induction and that was highly statistically significant.

That would likely suggest that a clinical decision on which agent to be used in clinical settings will be based on molecular apoptosis markers, liver functions and liver histopathology tests. In case of deteriorated liver biopsy histopathological score and declined liver function tests hydroxychloroquine would be preferred over simvastatin in combination to sorafenib based on its superior hepatoprotective and ameliorative results. This suggestion is supported by the work of other researchers mentioned before [54–57].

In case of HCC poor apoptotic response demonstrated in liver biopsy immunostaining of death receptors and active caspase-3 apoptotic index simvastatin would be a favorable companion to sorafenib.

In a nutshell, the current study showed that autophagy modulators in combination with sorafenib cause regression of chemotherapeutic resistance marked by lower HIF-1α and VEGFa on the gene transcription level, suppressed NF-κB and Gal-1 protein expression, G2/M phase arrest, programmed cell death activation, and ameliorative liver function when compared to sorafenib alone. Our findings emphasize the significance of HIF-1α, VEGFa, NF-κB and Gal-1 signaling pathways in the implementation of novel treatment techniques against drug-resistant cancers; especially sorafenib-resistant ones. The present study suggests that both autophagic inhibitors and inducers are crucial candidates for clinical trials of HCC based on the promising results of in-vivo animal models.

## Methods

### Establishment of the HCC model

All animal studies were performed in accordance with the National Institutes of Health’s (NIH) ethical guidelines for the care and use of laboratory animals (NIH publication No. 85-23, revised 1985). The study was given approval by the Research Ethics Committee of Mansoura University’s Faculty of Pharmacy (#2022-71). Male Sprague-Dawley rats weighing 220 g (8–10 weeks old) were purchased from the “Medical Experimental Research Center,” Mansoura, Egypt. For two weeks, the rats were subjected to acclimatization under typical laboratory conditions of controlled room temperature (25 ± 2 °C) and a 12 h light/dark cycle. They were allowed free access to water and food. HCC was established in rats by a single 200 mg/kg body weight intraperitoneal (i.p.) injection of diethylnitrosamine (DEN; Sigma–Aldrich; St. Louise, MO, USA); two weeks later, an i.p. injection of 200 mg/kg body weight thioacetamide (TAA; Sigma–Aldrich) was administered twice per week for 16 weeks [5]. At the end of this period, the HCC model’s establishment was validated by a liver histopathological examination and an assessment of serum alpha feto protein (AFP) levels.

### Experimental design

Rats were divided into seven groups with no blinding done, as follows: Control group (N) which received no treatment (*n* = 8); HCC group in which rats had HCC but received no further treatment (*n* = 8); sorafenib group (SF) which received 10 mg/kg sorafenib tosylate (LC laboratories; Boston, MA, USA) everyday through oral gavage for three weeks (*n* = 8) [61, 62]; hydroxychloroquine group (CQ) which received 60 mg/kg CQ (plaquenil®; Sanofi Pharmaceutical Corporation, Paris, France) everyday through oral gavage for three weeks (*n* = 8) [63–65]; simvastatin group (SV) which received 10 mg/kg SV (granted by EVA Pharma Company for Pharmaceuticals; Cairo, Egypt) everyday through oral gavage for three weeks (*n* = 8) [66, 67]; sorafenib + hydroxychloroquine group (SF + CQ) which received a combination of 10 mg/kg sorafenib and 60 mg/kg CQ by oral gavage daily for 3 weeks (*n* = 8); and sorafenib + simvastatin group (SF + SV) which received a combination of 10 mg/kg sorafenib and 10 mg/kg SV by oral gavage daily for 3 weeks (*n* = 8). While no formal statistical methods were employed for sample size estimation, a rationale for the chosen sample size of 8 animals per experimental group is provided based on relevant literature and practical considerations [68]. Additionally, no method of randomization was used.

### Sample collection

At the end of the experiment, rats were starved for 12 h and allowed free access to water. Blood samples were drawn through retro-orbital puncture and centrifuged for serum separation, however it would be prudent to withdraw blood samples from heart as results would be more representative. Instantly after sacrificing the rats, the liver was isolated and divided into four sections. The first section was put into RNA-later (Qiagen-Germany) and flash frozen in liquid nitrogen to measure gene expression by qPCR. The second section was cut into pieces, flash frozen in liquid nitrogen and then stored at −80 °C for western blotting and cell cycle analysis. The third section was preserved in a primary fixative solution of 2.5% glutaraldehyde +2% paraformaldehyde containing 0.1 M sodium phosphate buffer pH 7.4 at 4 °C for 24 h for transmission electron microscopy (TEM) analysis. The last section was fixed in neutral formalin, embedded in paraffin blocks and histopathologically examined. The formula {Liver index = [Liver weight (g)/Body weight (g)] × 100} was used to calculate liver index [69].

### Biochemical analysis

Serum kinetic colorimetric analysis of alanine aminotransferase (Spectrum Diagnostics, Egypt), gamma-glutamyl transferase (Human diagnostics, Germany), alkaline phosphatase (Human diagnostics, Germany) activities, and also, for endpoint colorimetric assays to measure the levels of albumin (Biodiagnostic, Egypt) and total bilirubin (Diamond Diagnostics, Egypt).

### Histopathological examination of hepatic tissues

After being fixed in formalin, liver tissues were embedded in paraffin blocks, dissected at a 4 μm-thickness to be stained with hematoxylin and eosin (H&E) and Masson’s trichrome for histopathological examination under a light microscope. H&E-stained hepatic sections were used to evaluate necroinflammatory scores guided by Ishak modified Histology activity index [70]. In H&E-stained liver sections, necroinflammatory alterations were estimated as the total of five categories: periportal or periseptal interface hepatitis (0–3), necrosis (0–3), ballooning degeneration (0–4), steatosis (0–3) and fibrosis (0–3). As for the measurement of tumor nodule size, the mean diameter of each nodule (in micrometers square) was calculated by measuring two diameters perpendicular to each other.

For semi-quantification of collagen fiber deposits in hepatic tissues, liver sections were stained with Masson’s trichrome [71], and the fibrosis degree was assessed using Image J software. The mean ± standard error was used to express the degree of central vein thickening, portal area expansion, and parenchymal fibrous regions in each of the ten fields. The mean percentage of the three zones was employed to compute the overall fibrotic area for each sample.

### Immunohistochemical analysis

Immunohistochemical analysis of p62, nuclear factor kappa B (NF-κB), active caspase-3, Tumor Necrosis Factor-alpha (TNF-α), FAS (also known as CD95) & TNF-Related Apoptosis-Inducing Ligand (TRAIL) were carried out. Primary antibodies against p62 (ABclonal, USA, dilution 1:200, catalog# A11250), NF-κB (Bioss, USA, dilution 1:400, catalog# bs-20159R), cleaved caspase-3 (Servicebio, China, dilution 1:1000, catalog# GB11532), TNF-α (Servicebio, China, dilution 1:500, catalog# GB11188), FAS (Elabscience, USA, Dilution 1:400, catalog# E-AB-70336) and TRAIL (abcam, UK, dilution 1:100, catalog# ab231063) were used in accordance with the standard protocols. Succinctly, 5 μm thick hepatocyte sections were deparaffinized with xylene, and then rehydrated with a graded ethanol concentration (100-95-75%). The Heat Induced Epitope Retrieval approach was used to retrieve epitopes utilizing Marque triology and a pressure cooker. Heating in citrate buffer pH 6 improved antigen recovery after five changes of distilled water and phosphate buffered saline (PBS). Hydrogen peroxide (3%) was employed to inhibit endogenous peroxidase activity. Following that, the tissue was incubated for 1 h with the matching antibodies; then with Ultra Vision horseradish peroxidase (HRP) polymer for 15 min. Each slide was covered with a previously prepared 3,3’Diaminobenzidine (DAB) substrate solution. The solution was made up of a 1:1 mixture of DAB chromogen solution and DAB buffer solution. The final three steps were distilled water washing, hematoxylin counter-staining, and dehydration using xylene and escalating degrees of ethanol. The slides finally were visualized under a light microscope. Images were analyzed by Image J software to calculate stain intensity by the area fraction method.

### Calculation of apoptotic index

Apoptotic index was calculated as the number of apoptotic cells per 10 high power fields [72].

### Autophagy assessment

There is presently no “gold standard” for assessing autophagic activity that can be used in all experimental settings. Moreover, evaluating autophagic flux in vivo or in organisms is currently one of the poorest explored topics, and optimum approaches might not exist. This research used four fundamental techniques for detecting macro-autophagy. We proposed three alternative ways for analyzing autophagy that are extensively utilized. To assess autophagic flux, the following approaches were used: immunoblotting of p62 and microtubule-associated protein 1 light chain 3 (LC3) proteins. Also, we analyzed gene expression of LC3B and p62 via qPCR, and eventually, visualization of various autophagic bodies using TEM was performed [73].

### Transmission electron microscopy

Tissues were washed three times with 0.1 M sodium phosphate buffer +0.1 M sucrose. Secondary fixations were achieved in 2% sodium phosphate buffered osmium tetroxide, pH 7.4, at room temperature in a rotator for 90 min. Tissues were washed three more times with 0.1 M sodium phosphate buffer. Samples were dehydrated by treating twice with an increasing ethyl alcohol gradient series (30, 50, 80, 90, 96, and 100%) and were washed with acetone for 15 min twice. They were treated with an acetone-Epon mixture with increasing Epon concentrations (acetone:Epon 2:1, then 1:1, then 1:2) for 30 min each, and finally stored at 4 °C overnight in Epon pure solution. In new fresh Epon solution, samples were incubated for polymerization at 70 °C for 24 h.

Ultrathin sections were obtained from blocks by ultramicrotome (PowerTome by RMC Boeckeler, USA) set to 50–100 nm thickness. Sections were rinsed on 300 mesh copper grids and post-contrasted for 10 min with 8% uranylacetate, and for 5 min with 1% lead citrate [74]. After drying for ~15 min, ultrathin sections were observed at 160 kV using a JEOL JEM-2100 (Tokyo, Japan) at the EM Unit, Mansoura University, Egypt. Images were acquired with a Gatan-211.1404.0 system (CA, USA).

### Cell cycle analysis by flow cytometry

Tribukait method was used for preparation of liver samples [75]. In brief, cells were suspended in isotonic saline and passed through a 40–50 mesh counts/cm nylon mesh. Finally, isotonic Tris EDTA buffer solution (Tris 0.1 M, NaCl 0.07 M and EDTA 0.005 M, pH 7.5) with RNase (1 mg/ml) was added to cells. The cell suspension was centrifuged at 1800 rpm for 10 min, and the supernatant was aspirated. Pepsin (0.5% pepsin solution, pH 2.0) incubation at 37 °C for 10 min was used to obtain single cell suspension.

The cell suspension (1 mL) was processed in a Falcon 12 × 75 mm polystyrene tube. Samples were stained with propidium iodide by suspension in 1 ml of the staining solution for 30 min at 4 °C [76, 77]. For determination of subG1 phase, the stained cells were kept in dark for at least 24 h. The specimens were thereafter assessed using an accuri™ C6 BD flow cytometer (Biosciences, San Jose, CA, USA) with the accuri™ C6 software for analysis and data recording.

### ELISA assessment of serum galectin-1 and AFP

Serum galectin-1 (Gal-1) protein expression was assessed using a commercially available ELISA Kit (Bioassay, England, # Catalog No E2580Ra) according to the manufacturer’s instructions. Serum AFP protein expression was assessed using a commercially available ELISA Kit (precheck, USA, # Catalog No. 37312C) according to the manufacturer’s instructions.

### qPCR

Tissue samples (50 mg) were used for RNA extraction by the GeneJET RNA purification kit (Thermo Fisher Scientific, USA) following the instructions of the manufacturer for total RNA extraction. Then, gene expression analysis of Bcl2, vascular endothelial growth factor (VEGF), caspase-3, caspase-8, p62 and hypoxia-inducible factor-1-alpha (HIF-1α).

Genomic DNA was seized from the RNA samples by DNase 1, RNase-free kit (Thermo Fisher Scientific, USA). The purity and concentration of the extracted RNA were quantified on a Nanodrop 2000 UV–Vis spectrophotometer (Thermo Fisher Scientific, USA). RNA samples were reverse transcribed to complementary DNA (cDNA) according to the instructions of the High capacity cDNA reverse transcription kit (Thermo Fisher Scientific, USA) using the PCR Thermal cycler TCA0096 (Thermo Fisher Scientific, USA). Design of specific PCR primers was carried out using Refseq-RNA PubMed primer blast, primer3 and Netprimer websites in accordance with the gene sequence from PubMed (Entrez Gene). The designed primers were blasted using the NCBI/BLAST tool to ensure their specificity to the target gene (Table 2). RT-PCR reactions were carried out with the aid of SensiFast™ SYBR®-No ROX kit (Bioline, USA) on the real time PCR system StepOne Plus™, (Applied Biosystems, Thermo Fisher Scientific, USA.) according to manufacturer’s instructions. The housekeeping gene used was rat glyceraldehyde 3-phosphate dehydrogenase (GAPDH). Ct values for gene samples were normalized against GAPDH as internal control and relative gene expression was calculated following the ΔΔCt method.

**Table 2:** Sequences of qPCR primers.

Gene of interest	Primer sequence		Accession number	Product size	Reference
Caspase-3	Forward	5′-GGAGCAGTTTTGTGTGTGTGA-3′	NM_012922.2	191	[54, 68]
	Reverse	5′-TGTCTCAATACCGCAGTCCA-3′			
Caspase-8	Forward	5′-CCTTTCTCCTCCCTCTGACCTC-3′	NM_022277.1	193	[54]
	Reverse	5′-GTAACCTGTCGCCGAGTCCC-3′			
Bcl-2	Forward	5′-AGGATAACGGAGGCTGGGATG-3′	NM_016993.1	179	[54]
	Reverse	5′-TATTTGTTTGGGGCAGGTCT-3′			
LC3b	Forward	GCTCCATGCAGGTAGCAGGAA	NM_022867.2	102	[80]
	Reverse	AGCTCTGAAGGCAACAGCAACA			
p62	Forward	5‘-GGAGCAGTTTTGTGTGTGTGA -3	NM_175843.4	100	–
	Reverse	5’- GGGGGCACAGTGAATGATAAG -3’			
VEGFa	Forward	5’ TTTTGCTTCCTATTCCCCTCT – 3’	NM_001110333.2	150	–
	Reverse	5’ – TCTCTCTCTCTCTCTCTTCCTTGA – 3’			
GAPDH	Forward	5’ TCCCATTCTTCCACCTTTGA -3’	NM_017008.4	109	–
	Reverse	5’ – CCACCACCCTGTTGCTGTAG – 3’			
HIF1α	Forward	5’ – AGCAACTAGGAACCCGAACC – 3’	NM_024359.2	112	–
	Reverse	5’–AGAGAAAGGGGCAAGTCCAG – 3’			

*LC3B* microtubule-associated protein 1 light chain 3-B, *VEGFa* Vascular Endothelial Growth Factor a, *HIF1-α* Hypoxia inducible factor-1α.

### Western blotting of LC3I/II

Liver tissues were first pre-washed in PBS and homogenized in 5 mL pre-cooled lysis buffer (pH 7.4: 1% Triton X-100, 25 mM EDTA, 10 mM Tris-Base, 50 mM NaCl, 25 mM EGTA, 1% NP-40,) with phosphatase/protease inhibitors (1:1000; Sigma, MO, USA). Initially, Pierce™ 660 nm assay kit (Thermo Scientific, USA) was employed for total protein assessment. Sodium dodecyl sulfate (SDS) was used as a loading buffer. SDS buffer was heated with 30 µg of protein for 5 min at 96 °C. Then samples were loaded, separation by electrophoresis was performed (Cleaver Scientific Ltd, UK), and semi-dry Electroblotter (Bio-Rad, CA, USA) was used for sample transfer to PVDF membrane. Bovine serum albumin (5% in Tris-buffered saline-Tween-20) was used for protein blocking at room temperature. Finally, the membrane was stained at 4 °C for 18–20 h with 1:2500 β-actin (# A5060, Sigma, St. Louis, MO, USA) and 1:1000 rabbit anti-rat LC3 (#4108, Cell Signaling Technology, MA, USA). After washing, membranes were stained for 1 h at room temperature using 1:1000 HRP-conjugated goat anti-rabbit immunuglobulin as a secondary antibody (Dako, Glostrup, Denmark, # Catalog No P0448) [78]. For visualization, the membrane was incubated for 1 min in Western Lightning Plus ECL Chemiluminescence Reagents (Perkin Elmer, Waltham, MA) and images were taken captured using the Chemi-Doc imager (Bio-Rad, Hercules, CA, USA). The intensities of bands were determined and compared using the bands of β-actin bands as internal control [79].

### Statistical analysis

Every outcome was expressed as mean ± S.E.M. All the data met the assumption of normal distribution. The variance similarity between compared groups was confirmed using Bartlett’s test. Experimental results were examined using one-way analysis of variance (ANOVA) followed by Tukey’s post-hoc test. Necroinflammatory scoring results were analyzed using the non-parametric Kruskal–Wallis test followed by Dunn’s post-hoc test. Statistical tests were performed by GraphPad Prism 2019 v8.0.2.263 software (IBM Corp., Armonk, NY, USA). Statistical significance was considered at *p* < 0.05.

### Supplementary information

Western blot raw data (uncropped blot)

## Data Availability

The datasets generated and analyzed during the current study are available from the corresponding author on reasonable request.
