# Supplementary figures and images for: Autophagy modulation attenuates sorafenib resistance in HCC induced in rats
Source: Cell Death Dis. 2024 Aug 16;15(8):595. doi: 10.1038/s41419-024-06955-5 (PMC11329791; doi:10.1038/s41419-024-06955-5)

14 KDa

16 KDa

**LC3-I**

**LC3-II**

Uncropped membrane


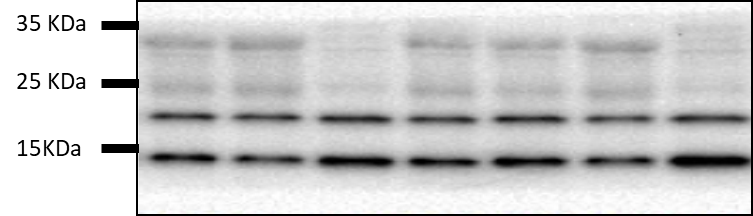

Supplement: Supplementary file 1 — Western blot raw data (uncropped blot) [file 41419_2024_6955_MOESM1_ESM.docx]
